# Evolution and Strengthening Effects of the Heat-Resistant Phases in Al–Si Piston Alloys with Different Fe/Ni Ratios

**DOI:** 10.3390/ma12162506

**Published:** 2019-08-07

**Authors:** Fanchao Meng, Yuying Wu, Kaiqi Hu, Yang Li, Qianqian Sun, Xiangfa Liu

**Affiliations:** 1 Key Laboratory for Liquid-Solid Structural Evolution and Processing of Materials, Ministry of Education, Shandong University, Jinan 250061, China; 2 Shandong Al & Mg Melt Technology Company Limited, Jinan 250061, China

**Keywords:** Al–Si alloys, heat-resistant phases, microstructure, mechanical properties

## Abstract

The evolution of three major heat-resistant phases (δ-Al_3_CuNi, γ-Al_7_Cu_4_Ni, T-Al_9_FeNi) and its strengthening effects at high temperature in Al–Si piston alloys with various Fe/Ni ratios were studied using field emission scanning electron microscope (FE-SEM), electron probe microanalysis (EPMA), and X-ray diffraction (XRD). With the increase of Fe/Ni ratios, the heat-resistant phases begin to evolve in category, morphology, and distribution. The results show that a suitable Fe/Ni ratio will cause the T-Al_9_FeNi phase to appear and form a closed or semi-closed network with δ-Al_3_CuNi and γ-Al_7_Cu_4_Ni phases instead of the originally isolated heat-resistant phases. As a result, the ultimate tensile strength of the optimized alloy reached 106 MPa with a Fe/Ni ratio of 0.23, which was 23.3% higher than that of base alloy at 350 °C, which is attributed to the fact that a closed or semi-closed network microstructure is advantageous to the bearing of mechanical loads. This work may provide useful ideas for the development of high temperature resistant piston alloys.

## 1. Introduction

Al–Si multicomponent piston alloys have been widely used in automobile manufacturing, such as in automobile engine pistons, owing to good castability, high elevated-temperature strength, light weight, good wear resistance, and low thermal expansion [1,2,3]. Recently, in order to meet the requirements of energy saving and emission reduction, the continuous improvement of engine efficiency has caused the working load of the piston to increase significantly, which puts higher requirements on the high temperature performance of the piston [4,5]. Therefore, researchers are paying more attention to the elevated-temperature properties of Al–Si piston alloys [6,7].

A lot of work has been done to improve the elevated-temperature performance of Al–Si piston alloys in the past decades. Alloying is the most effective method to improve the elevated-temperature properties of the Al–Si piston alloys by adding the transition elements such as Cu, Ni, Fe, and Mn. Li et al. [8] and Yang et al. [9] studied the changes in the category and morphology of heat-resistant phases with the addition of different Cu content, and the effect of heat-resistant phases at elevated-temperature on the properties of Al–Si alloys. Feng et al. [10] investigated the effects of thermal stable ε-Al_3_Ni phase content on Al–Si microstructure, mechanical properties and low cycle fatigue at 350 °C. Qian et al. [7] added Mn from 0.04% to 0.4% into ZL109 piston alloy and found that the solubility of Mn in dendritic Al_9_FeNi phase plays an important role in strengthening alloys. Shaha et al. [11] also reported the high temperature tensile properties of the Al–Si–Cu–Mg alloy, with additions of Mn and Mo. A lot of research has been done on the role of adding Fe element on the mechanical properties in Al–Si alloys. Abouei et al. [12] and Warmuzek [13] reported that adding Fe is an effective way to enhance the elevated-temperature properties of the alloy. It is well accepted that the Fe-rich phases are prone to a three-dimensional platelet form, such as the β-Al_7_Cu_2_Fe phase [14], which can induce stress accumulation during deformation [15,16]. To neutralize the harmful effects of Fe, it is a common method to add Mn, Cr, and Co elements to modify the β-Fe phase to α-Fe phase [12,17,18,19]. However, the above measures are limited in the improvement of the high temperature performance of Al–Si piston alloys. Moreover, there are three heat-resistant phases, Al_3_CuNi, Al_7_Cu_4_Ni, and Al_9_FeNi, which also play an important role in Al–Si piston alloys [20,21]. Few studies have focused on changing the ratio of Fe/Ni to adjust the morphology of Fe-bearing phases and the evolution of Ni-containing phases and the elevated-temperature tensile properties without adding Mn, Cr, and Co.

In this study, the evolution of heat-resistant phases and the elevated-temperature tensile properties of different Fe/Ni ratios were investigated in Al–Si–Cu–Mg–Ni–Fe alloys. Additionally, the influence of microstructure on high temperature tensile properties was also discussed.

## 2. Materials and Methods

The multicomponent Al–Si–Cu–Mg–Ni–Fe piston alloys were prepared using 99.85% (all compositions cited herein are in wt.% unless otherwise stated) commercial purity Al (), 98.5% commercial purity crystalline Si (), 99.9% purity Cu (), Ni (), Mg () and Fe (), and all the above alloys were provided by Shandong Al & Mg Melt Technology Co. Ltd. (Jinan, China). In this work, the high temperature properties of several alloys with similar composition were compared. The changes of several alloy compositions were achieved by changing the mass ratio of Fe and Ni, the chemical compositions of the alloys were measured by spectrometer (MAXx, Spectro Kleve, Germany), which are listed in Table 1. The alloy ingots were melted in a graphite crucible (20#, Major Sheng, Foshan, China) by a medium frequency induction furnace at about 800 °C. The temperature was measured by a digital chromel-alumel thermocouple (WRNK-181, Pulian, Shanghai, China). When the temperature of the melts kept steady at approximately 750 °C, the refining process of the melts were carried out by using C_2_Cl_6_ (Huasai, Jinan, China) to remove slag and gas. After that, the melts were transferred to an electric resistant furnace (SG2-12, Longyi, Longkou, China) and held at 760 °C for 30 min to refine primary silicon with 0.6 wt.% of an Al-P master alloy, which was provided by Shandong Al & Mg Melt Technology Co. Ltd. (Jinan, China). The melts at 760 °C were finally poured into the metal mold (Gravity casting, Yingshun, Yongkang, China), which was preheated to 200 °C, to get tensile test bars. The above operations were repeated several times to obtain different tensile test bars by changing the corresponding Fe/Ni ratios, respectively.

All test bars were heat-treated after being processed into a “dog bone” type sample, and the examined alloy was subjected to solution treatment and artificial aging, i.e., T6 to obtain higher mechanical properties. The specific regulation was solution treated at 520 °C for 2 h; water quenched; aging treated at 180 °C for 8 h and cooled in air. Then, the tensile test was conducted with a strain rate of 2 mm/min with the help of an extensometer after the samples were isothermally held for 30 min at 350 °C using a WDW-100D universal material testing machine, which was provided by Jinan Zhongzheng Testing Machine Manufacturing Co. Ltd. (Jinan, China. The tensile strength data of each alloy illustrated in Table 1 is an average of four tensile specimens.

Specimens for metallographic microstructure analysis were cut from tensile bars and a portion of the sample was deep-etched with a concentration of 10% NaOH (Yizhong, Tianjin, China) for 5 min. Samples for fracture microstructure analysis were taken from the test bars after testing. X-ray diffractometer (XRD, D/max-rB, Rigaku, Tokyo, Japan) with a copper target as anode was conducted and analyzed with software Jade (Jade5, MDI, California, USA). The microstructures of the specimens were observed by the field emission scanning electron microscope (FESEM, SU–70, Hitachi, Tokyo, Japan) with an energy dispersive spectroscopy (EDS, EX-250, Horiba, Kyoto, Japan) detector at 15 kV. The microstructural compositions were also characterized by electron probe microanalysis (EPMA) (JXA-8530F Plus, Hitachi, Tokyo, Japan) combined with a wavelength dispersive spectroscopy (WDS) system manufactured by OXFORD. For examined mapping, the acceleration voltage was 15 kV and the probe current was 99.9 nA. As for the X-ray lines, the Ka1 lines were used for Al, Si, Ni, Cu, Fe, and Mg. The characteristics of the intermetallic compounds were extracted and calculated from the carefully prepared secondary electron images by ImageJ software (Image 1.40g, National Institutes of Health, Bethesda, USA), based on the different contrast intensities [8].

## 3. Results and Discussion

### 3.1. Identification and Evolution of the Heat-Resistant Phases

**Figure 1:**
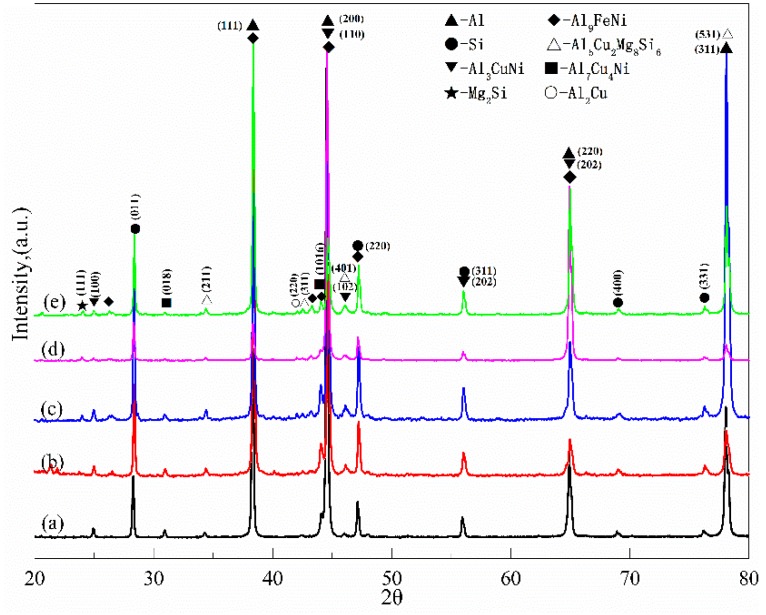
X-ray diffractometer (XRD) patterns of Al-12Si-4Cu-1Mg alloys with different Fe/Ni ratios: (**a**) 0.13% Fe and 2.55% Ni (Fe/Ni = 0.05), (**b**) 0.30% Fe and 2.63% Ni (Fe/Ni = 0.11), (**c**) 0.47% Fe and 2.54% Ni (Fe/Ni = 0.19), (**d**) 0.62% Fe and 2.68% Ni (Fe/Ni = 0.23), (**e**) 0.80% Fe and 2.62% Ni (Fe/Ni = 0.31).

The intermetallic compounds are the main heat-resistant phases at elevated-temperature in Al–Si piston alloys [7]. Figure 1 shows the XRD patterns of the tested alloys with different Fe/Ni ratios. The Q-Al_5_Cu_2_Mg_8_Si_6_ phase and the two kinds of Ni-bearing phases, including the δ-Al_3_CuNi phase and the γ-Al_7_Cu_4_Ni phase, were detected in alloys A, B, C, D, and E. From the XRD patterns, it can be seen that Fe-bearing phases begin to appear when the ratio of Fe/Ni reaches 0.11 (0.30% Fe and 2.63% Ni). Additionally, the peaks of Mg_2_Si and Al_2_Cu have also been observed; this phenomenon has also been discovered by other researchers [8,22].

**Figure 2:**
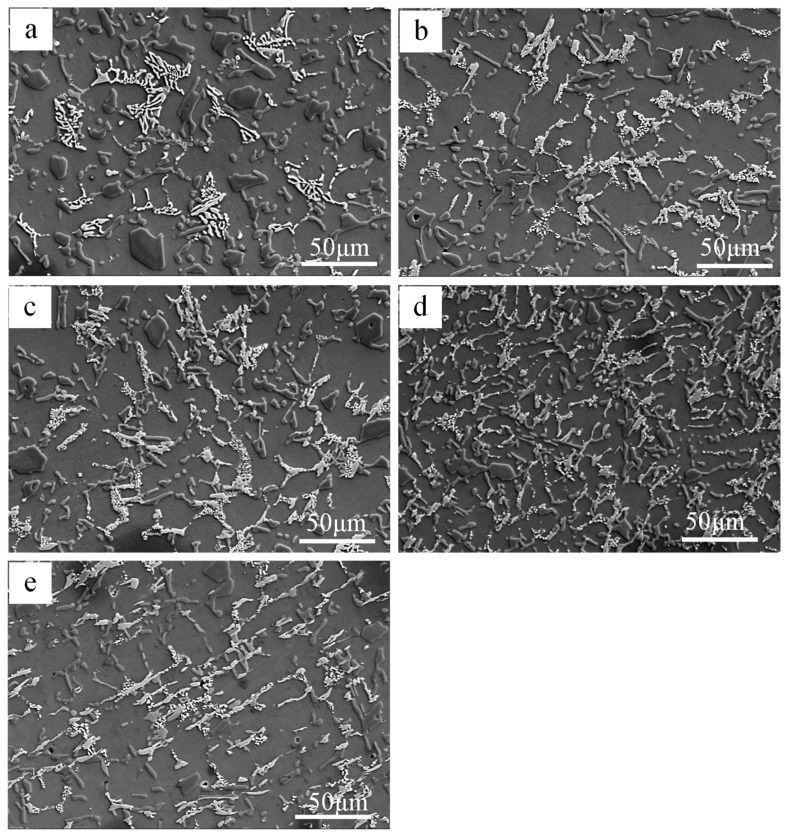
SEM images of Al-12Si-4Cu-2.5Ni-1Mg-xFe alloys after T6 treatment with different Fe contents: (**a**) Alloy A, (**b**) alloy B, (**c**) alloy C, (**d**) alloy D, (**e**) alloy E.

**Figure 3:**
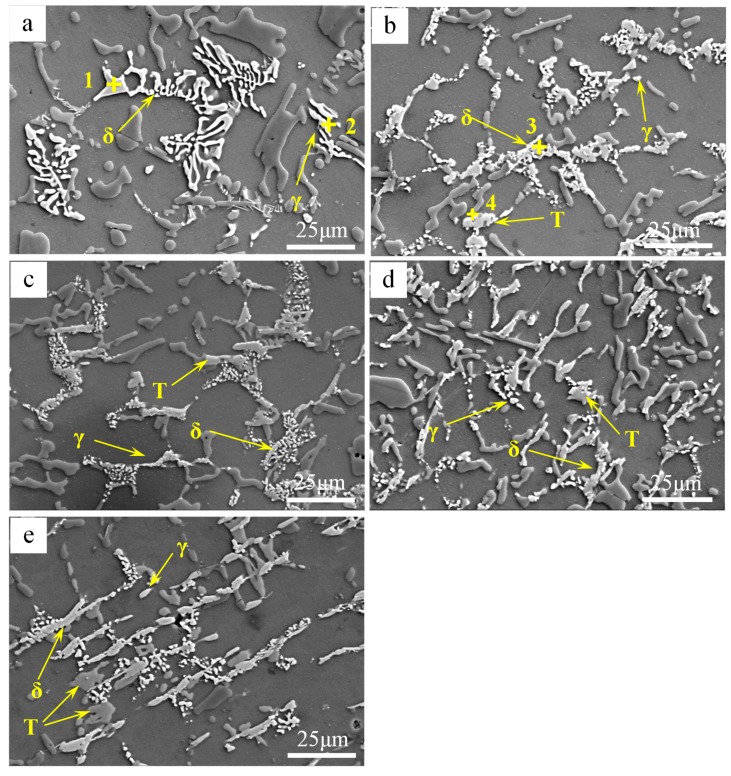
SEM images of intermetallic compounds: (**a**) Alloy A, (**b**) alloy B, (**c**) alloy C, (**d**) alloy D, (**e**) alloy E. (δ: Al_3_CuNi phase, γ: Al_7_Cu_4_Ni phase, T: Al_9_FeNi phase).

Figure 2 shows the SEM results of the main phases in tested alloys at the T6 state. Figure 3 shows a partial enlarged image of Figure 2 and the main heat-resistant phases in the examined alloy are identified by EDS and the specific results are listed in Table 2. In alloy A, it can be found in Figure 2a that numerous skeleton-like phases are present in the Al matrix. The skeleton-like phase is identified as the δ-Al_3_CuNi phase with the help of EDS and the δ-Al_3_CuNi phase is larger in size and more isolated in the matrix. At the same time, a small quantity of the γ-Al_7_Cu_4_Ni phase with a short rod-like can be observed, as can be seen in Figure 2a and Figure 3a.

Further increasing the Fe/Ni ratio, three main heat-resistant phases, i.e., δ-Al_3_CuN, γ-Al_7_Cu_4_Ni, and T-Al_9_FeNi phases, simultaneously appear in alloys B, C, D, and E, as can be seen in Figure 2b–e. However, when the Fe/Ni ratio adds up to 0.11, the δ-Al_3_CuNi phase with a skeleton-like morphology begins to decrease and the bulk T-Al_9_FeNi appears, as shown in Figure 2b. As shown in Figure 2c, three main heat-resistant phases present as an annular or semi-annular shape in alloy C. With the addition of the Fe/Ni ratio to 0.23, the δ-Al_3_CuNi, γ-Al_7_Cu_4_Ni, and T-Al_9_FeNi phases are distributed in alloy D in the form of semi-reticular morphology. It is worth noting that the δ-Al_3_CuNi phase exhibits a strip-like morphology in alloy E with a Fe/Ni ratio of 0.31, as shown in Figure 2e and Figure 3e. When the Fe/Ni ratio exceeds 0.05, the composition of the δ-Al_3_CuNi phase also changes, the composition shown in spectrum 3 of Table 2 contains a small amount of Si and Fe elements. The change of composition of the δ-Al_3_CuNi phase may be caused by the dissolution of Si and Fe elements [13]. Previous studies have shown that the δ-Al_3_CuNi phase can dissolve 3 at.% Fe [8] and 2.7 at.% Si [23]. Moreover, the Si and Fe elements in the δ-Al_3_CuNi phase may also come from other adjacent phases. The Al_9_FeNi phase contains a small amount of Si, as shown in spectrum 4 of Table 2; many researchers have also found this phenomenon [13,24,25].

**Figure 4:**
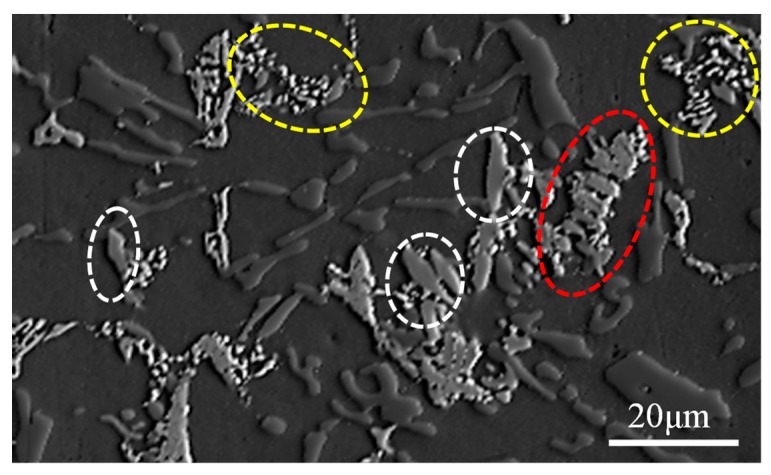
The SEM micrograph of alloy D for phase identification using EPMA (red circle: δ-Al_3_CuNi; white circle: T-Al_9_FeNi; yellow circle: δ-Al_3_CuNi).

**Figure 5:**
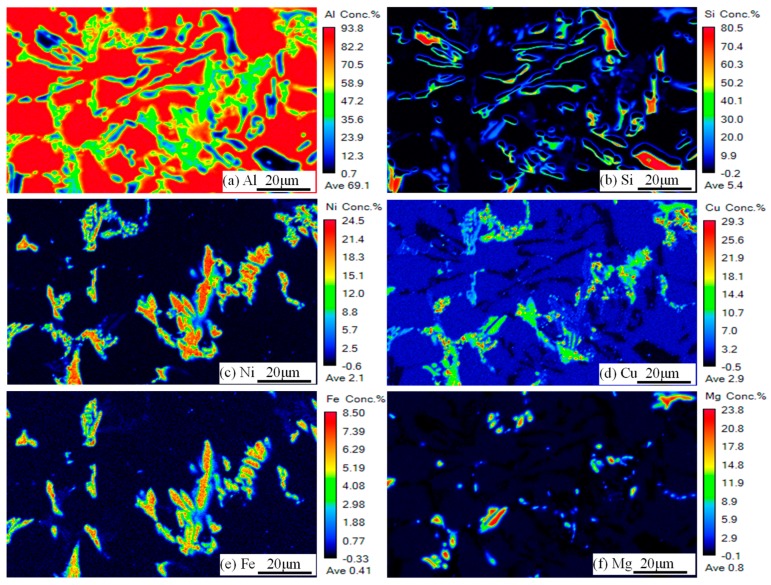
(**a**–**f**) The EPMA mapping results of components Al, Si, Ni, Cu, Fe, Mg at alloy D corresponding to Figure 4.

Alloy D has the highest ultimate tensile strength (as seen in Table 1), its microstructural composition typically reflecting the change in composition caused by the addition of Fe. To further verify the phase category in the tested alloy, Figure 4 shows an SEM photograph of alloy D corresponding to Figure 5 showing the EPMA mapping results In Figure 5a–e, it is observed that a high concentration of Al, Cu, Ni, Fe, and Si were concentrated in this region, as shown by the red dashed circle in Figure 4. Combined with the EDS results, this evolution that the δ-Al_3_CuNi phase transforms into a strip-like morphology due to the dissolution of Fe and Si elements was further verified. The blocky phase marked by the white dotted line and the skeleton-like phase marked by the yellow ellipse in Figure 4, respectively, correspond to the regions where Al, Fe, Ni and Al, Cu, Ni are concentrated in Figure 5, i.e., the T-Al_9_FeNi and δ-Al_3_CuNi phases. In general, the EPMA mapping results are consistent with the EDS results.

**Figure 6:**
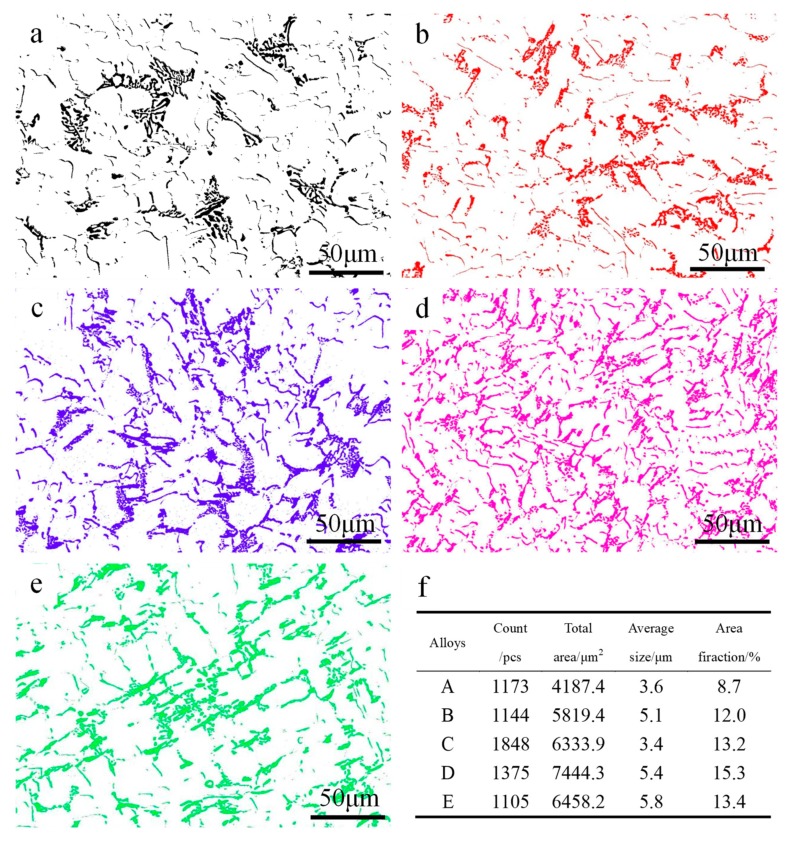
The extracted three heat-resistant phases after the T6 treatment: (**a**) Alloy A, (**b**) alloy B, (**c**) alloy C, (**d**) alloy D, (**e**) alloy E; (**f**) the characteristic values of the three main heat-resistant phases.

Figure 6 shows the changes in size, morphology, and distribution of the three main heat-resistant phases by ImageJ software. Because the three major heat-resistant phases have higher brightness, it is easy to extract them using ImageJ software. Three regions are randomly selected in the alloy to obtain the average characteristic values of the intermetallics. The results of the extraction and calculation are shown in Figure 6. It can be seen from Figure 6a,e that the fishbone heat-resistant phases and the strip-like heat-resistant phases are distributed on the matrix in isolation, which makes them unable to fully contribute to high temperature strength under high temperature load. As is apparent from Figure 6d, when the ratio of Fe/Ni is 0.23, the area fraction and number of the three main heat-resistant phases are maximized in the matrix, which also shows that the degree of connection between the heat-resistant phases is maximized. It can be deduced that the formation of closed or semi-closed network structures will make the most effective contribution to high temperature strength.

### 3.2. Elevated Temperature Tensile Properties and Microstructure Analysis

**Figure 7:**
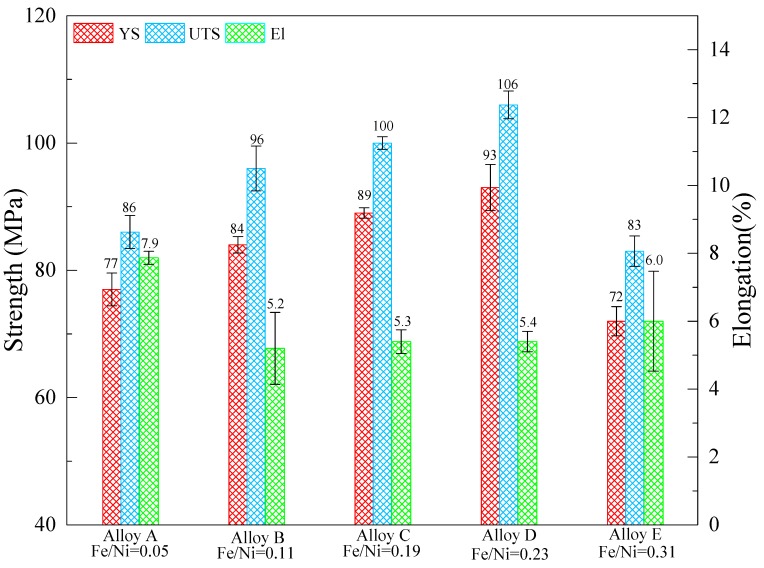
The tensile properties of Al-12Si-4Cu-1Mg alloys after T6 treatment with different Fe/Ni ratios at 350 °C (YS: yield strength; UTS: ultimate tensile strength; El: elongation).

Figure 7 shows the tensile properties on ultimate tensile strength (UTS), yield strength (YS) and elongation (El) of the Al-12Si-4Cu-1Mg alloys with different Fe/Ni ratios at T6 state, respectively. It can be seen that both the UTS and YS at 350 °C were dramatically increased with increasing of the Fe/Ni ratio within a certain range; with the addition of the Fe/Ni ratio from 0.05 to 0.23, the UTS and YS of alloy D were higher than that of alloy A by 20.8% and 23.3%, respectively. However, the El decreased from 7.9% to 5.4%. Noticeably, alloy E had the lowest elevated-temperature tensile strength which is even less than that of alloy A. The intermetallic phases are the main elevated-temperature heat-resistant phases in Al–Si alloys, its thermal stability plays an important role in the mechanical properties of cast Al–Si alloys [16]. In cast Al–Si multicomponent alloys, the thermal stability of Al_3_CuNi, Al_7_Cu_4_Ni, and Al_9_FeNi are about 350–400 °C [20,21,26,27]. In view of the small difference between the thermal stability among Al_3_CuNi, Al_7_Cu_4_Ni, and Al_9_FeNi, the elevated-temperature strength of the alloys mainly depends on the size, morphology, and distribution of the intermetallic phases. The eutectic grain boundaries are the weakest areas in alloy at elevated-temperature, this view is widely accepted by researchers [28,29,30]. At elevated-temperatures, the sliding of grain boundaries is impeded by the thermally stable intermetallic compound with the stress increasing.

**Figure 8:**
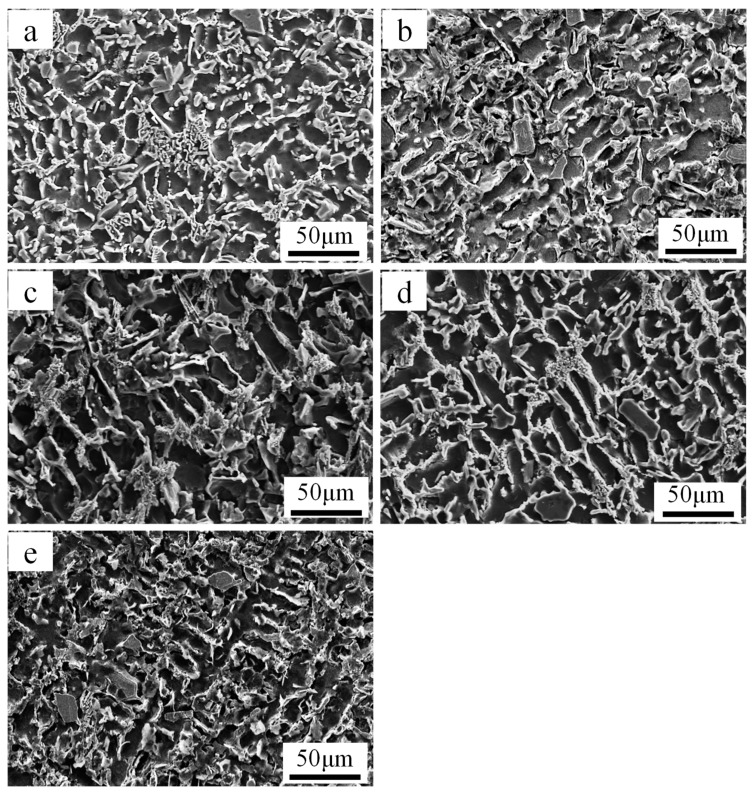
Scanning electron micrographs of deep etched Al-12Si-4Cu-1Mg alloys after T6 treatment with different Fe/Ni ratios: (**a**) Alloy A, (**b**) alloy B, (**c**) alloy C, (**d**) alloy D, (**e**) alloy E.

It can be seen from Figure 2 and Figure 6 that the connectivity of the heat-resistant phases becomes larger as the Fe/Ni ratio increases except for alloy E. The degree of interconnectivity of the rigid phases can result in an extra increase of load transfer from matrix to the reinforcement which is more important at high temperatures when the matrix gets soft [22,27]. The network structure can effectively strengthen the α-Al matrix by transferring the load from the α-Al matrix to the heat-resistant phases. At the same time, the thermally stable intermetallic framework can increase the critical stress of the crack initiation [15] and hinder the deformation of the α-Al matrix. So, the suitable size and high interconnectivity of the intermetallic compound provides more efficient contribution to the elevated-temperature strength. The morphology characterizations for the examined alloys with the help of ImageJ software reveal the reason why alloy D possesses the highest elevated-temperature strength among the tested alloys. The three major heat-resistant phases (δ-Al_3_CuNi, γ-Al_7_Cu_4_Ni, T-Al_9_FeNi) have the area fraction in alloy D compared with other tested alloys, as can be seen from Figure 6f. So, alloy D has the most effective volume utilization on three heat-resistant phases and has the most efficient contribution to the elevated-temperature strength. Besides, from the comparison of the morphology of alloy after being deep etched, as can be seen in Figure 8, it can be obviously seen that the degree of connection of the network structure formed by the reinforcement is significantly increased. A highly interconnected rigid phase network allows the alloy to withstand greater stresses prior to fracture, but because the heat-resistant phases are tightly connected, micro-cracks can propagate rapidly through the network, resulting in a decrease in plasticity. However, the heat-resistant phases become isolated and the connectivity between them deteriorates when the Fe/Ni ratio is increased to 0.31 in alloy E, which causes the high temperature strength of the alloy to decrease as opposed to the increase in the Fe/Ni ratio.

**Figure 9:**
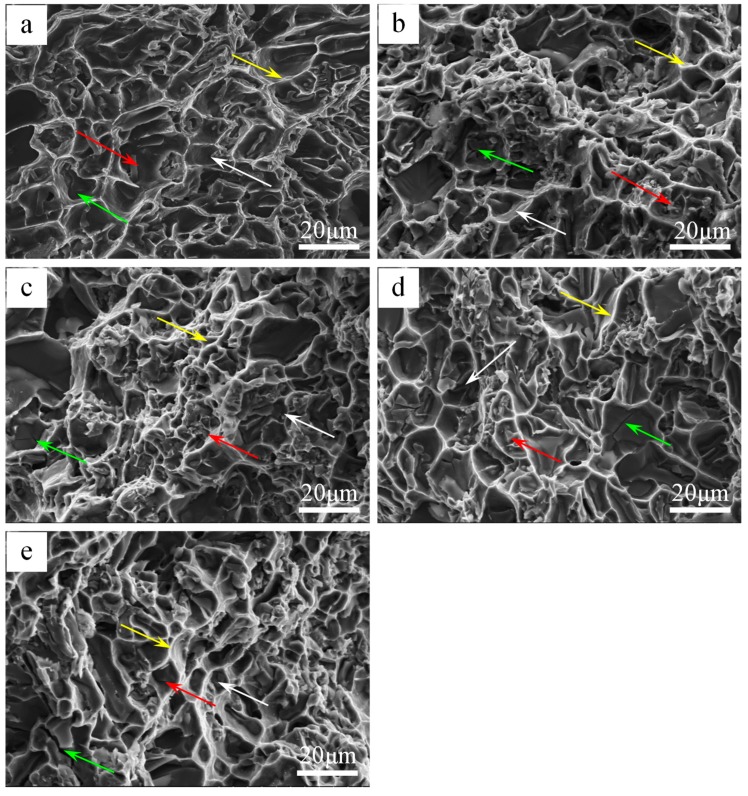
SEM micrographs of fractured surfaces on the tensile specimens at different Fe/Ni ratios (350 °C): (**a**) Alloy A, (**b**) alloy B, (**c**) alloy C, (**d**) alloy D, (**e**) alloy E.

To further investigate the failure mechanism of the castings at elevated-temperature, the fracture surface of each tested alloy was examined using SEM. Figure 9 shows the fractographs of the tensile samples at 350 °C. It can be observed that the fracture surface showed quasi-cleavage features along with many smooth flat areas separated by tearing ridges (as pointed by yellow arrow). As shown in Figure 9, many secondary cracks (green arrow) are formed due to the smash of primary Si in the matrix [11], which is owed to a strong interaction between the dislocation and hard particles. Figure 9 shows some of separated heat-resistant phase dendrites, as indicated by the red arrow. During the tensile test, the elastic deformation of α-Al grains is hindered by the heat-resistant phases, which result in stress concentrations along interfaces between the heat-resistant phases and α-Al grains. With the increase of tensile stress, the interface will first produce micro-cracks. It is worth noting that the heat-resistant phases play an important role in increasing the critical stress of the crack initiation [15]. The Fe/Ni ratio increases from 0.05 to 0.23, and the heat-resistant phases in the dimple (white arrow) increases significantly, which also explains the increase in high temperature strength. These heat-resistant phases will inhibit the movement of dislocations, which leads to a decrease in ductility.

## 4. Conclusions

The evolution of the heat-resistant phases and its strengthening effects on Al-12Si-4Cu-1Mg alloy with different Fe/Ni ratios at high temperature were studied. The conclusions can be summarized:

1. As the Fe/Ni ratio exceeds 0.05, the morphology of Al_3_CuNi changes from a skeleton-like to a strip-like morphology and the isolated distribution between the heat-resistant phases also disappears.
2. In the case where the Fe/Ni ratio is 0.19, the T-Al_9_FeNi phase appears as an annular or semi-annular distribution with other heat-resistant phases.
3. When the Fe/Ni ratio in the Al-12Si-4Cu-1Mg alloy is about 0.23, the heat-resistant phases exhibits a semi-reticular network distribution, and a significant improvement at high temperature strength can be obtained, the UTS at 350 °C reached 106 MPa, which is about 23.3% higher than the alloy with a Fe/Ni ratio of 0.05.

## Figures and Tables

**Table 1:** Chemical composition and ultimate tensile strength of Al-12Si-4Cu-2.5Ni-1Mg-xFe alloy in the T6 state (wt.%).

Alloys	Elements (wt.%)						Fe/Ni Ratios	UTS 350 °C (MPa)
	Si	Cu	Mg	Ni	Fe	Al		
A	12.22	3.89	0.94	2.55	0.13	Bal.	0.05	86
B	12.15	4.08	0.92	2.63	0.30	Bal.	0.11	96
C	12.16	3.95	1.01	2.54	0.47	Bal.	0.19	100
D	11.98	4.02	1.02	2.68	0.62	Bal.	0.23	106
E	12.07	4.06	0.99	2.62	0.80	Bal.	0.31	83

**Table 2:** Compositions of the intermetallic compound in Figure 3 as measured by EDS (at.%).

Spectrum	Al	Si	Cu	Ni	Fe	Identified Compounds
1	59.9	-	20.5	19.6	-	Al_3_CuNi
2	59.6	-	26.7	13.7	-	Al_7_Cu_4_Ni
3	67.3	5.5	12.1	12.3	2.8	Al_3_CuNi
4	81.0	1.8	-	12.6	4.6	Al_9_FeNi
